# Kimura's disease and its masquerade with a malignancy: A case report

**DOI:** 10.1016/j.amsu.2022.103864

**Published:** 2022-05-27

**Authors:** Fareeha Farooqui, Ibad ur Rehman, Sania Waseem, Irfan Ullah, Mehar Jehan, Muhammad Sohaib Asghar

**Affiliations:** a Shifa International Hospital, Islamabad, Pakistan; b Kabir Medical College, Gandhara University, Peshawar, Pakistan; c Institute of Public Health & Social Sciences (IPH&SS), Khyber Medical University, Peshawar, Pakistan; d Dow University of Health Sciences-Ojha Campus, Karachi, Pakistan

**Keywords:** Kimura's disease, Parotidectomy, Malignancy, Eosinophilia

## Abstract

Kimura's disease is an uncommon chronic condition that often masquerades as a malignancy. It is reported predominantly in males of South-East Asia in their second and third decade of life. Most of the patients present with increased eosinophilia, follicular hyperplasia and high levels of IgE circulating in their blood. In this case, we report a patient of 29 years age who presented with complaints of left parotid and left post-auricular swellings. It was painless and growing gradually in size over the period of 8 years. It was asymptomatic during the whole period of the disease. Patient was diagnosed on histology and subsequent immunohistochemistry reports. Treatment in this patient included parotidectomy and lymph node dissection. It was followed by local radiation to prevent recurrence. It can be concluded that Kimura's disease may vary in its presentation but a few features remain characteristic. No reports of malignant change have been reported as of yet making its prognosis quite favorable for the patient. Surgery remains the choice of treatment but for more effective approach, it should be followed by radiation.

## Highlights

• In a patient presenting with neck mass along with history of weight loss, Kimura's Disease should be suspected.
• Multidisciplinary approach is required for prompt diagnosis and treatment in such cases.
• More knowledge on Kimura's disease will help physicians in coming up with a better management plan in future.

## 1 Introduction and importance

Initially reported for the very first time in 1937 in China, Kimura's disease is a benign, indolent and usually a self-limiting condition that is often mistaken for malignancy [1,2]. The disease was known as “eosinophilic hyperplastic lymphogranuloma” till 1948 but later on its vascular component was observed by Kimura T and it was subsequently named Kimura's disease [2]. Only 200 worldwide cases of Kimura's disease were reported till September 2020 but the condition's exact prevalence is unknown [3]. Most of these cases have been reported in young Asian males in their early 20s. So far, no specific etiology has been identified for the condition [4]. Kimura's disease presentation has varied presentation according to the cases reported so far but a few features are consistent throughout, these include eosinophilia and raised IgE levels [3,4].

Here we report this case of a 29-year male diagnosed with Kimura's disease in accordance with the SCARE criteria [5], to add to the existing knowledge regarding the disease's presentation, how it may show in laboratory investigations and how it may be treated.

## 2 Case Presentation

A 29-year-old man, with no comorbidities presented to the surgical clinic with complaint of recurrent left parotid enlargement and left posterior auricular swelling forthe last 8 years. Initially, it was operated upon in a local setup almost 12 years back. Swellings had increased in size gradually over the years and were painless. They were not associated with pus discharge or bleeding. He had a history of weight loss of 8 kgs in 8 months but had no complaints of fatigue, fever, or any associated muscular weakness. On physical examination, there was a visible, firm and non-tender swelling at the angle of the jaw. The overlying skin looked erythematous and had tiny pustules over it. There were multiple post auricular swellings. Systematic examination was normal. A set of baseline investigations were ordered as listed in Table 1.

**Table 1:** Baseline Investigations of patient (pre-operative).

Hematology		
Investigation	Results	Reference value
WBC Total	4760/μl	4000 μl–12,000 μl
RBC Total	5.14 m/μl	(4.5–6.5) m/μl
Hemoglobin	15.3g/dl	(13.5–18.0)g/dl
Platelet count	159000	(150,000–400,000) μl
Neutrophils	42%	(54–62)%
Lymphocytes	28%	(25–33)%
Monocytes	8%	(1–4)%
Eosinophils	22%	(1–3)%
**Chemistry**		
HBs Ag	Reactive	
Hepatitis C virus Ab	Non-reactive	
Creatinine	0.70mg/dl	0.72–1.25 mg/dl

**Fig. 1:**
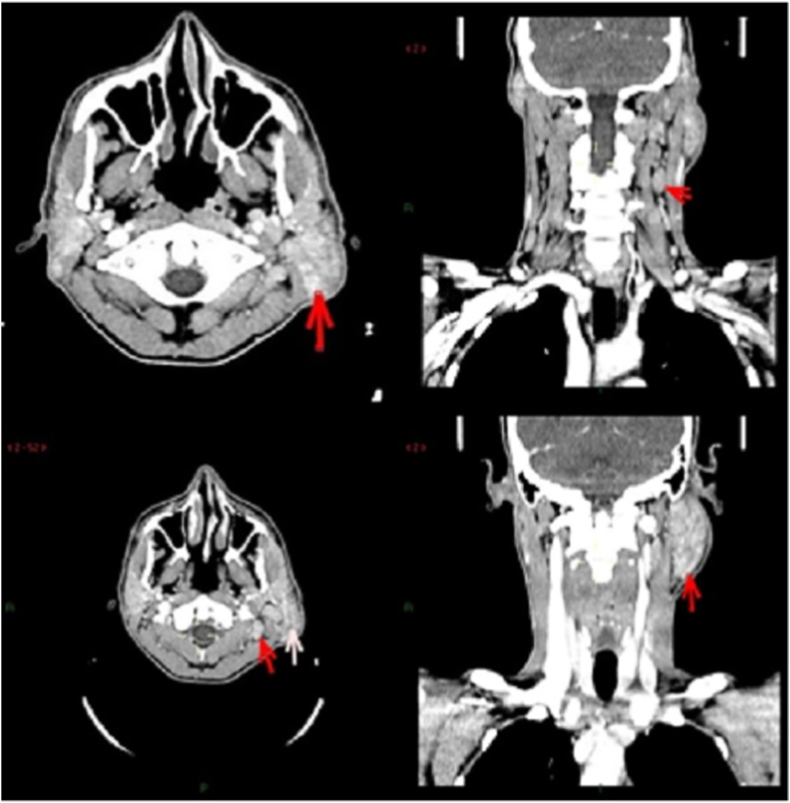
CT scan of patient pre-operatively.

Contrast-enhanced computerized tomography (CT) scan of the neck showed a mildly enlarged left parotid gland (5.8cm in anteroposterior dimension) showing heterogeneous enhancement with intraparotid mildly enlarged and prominent lymph nodes along with cervical lymphadenopathy (Fig. 1).

**Fig. 2:**
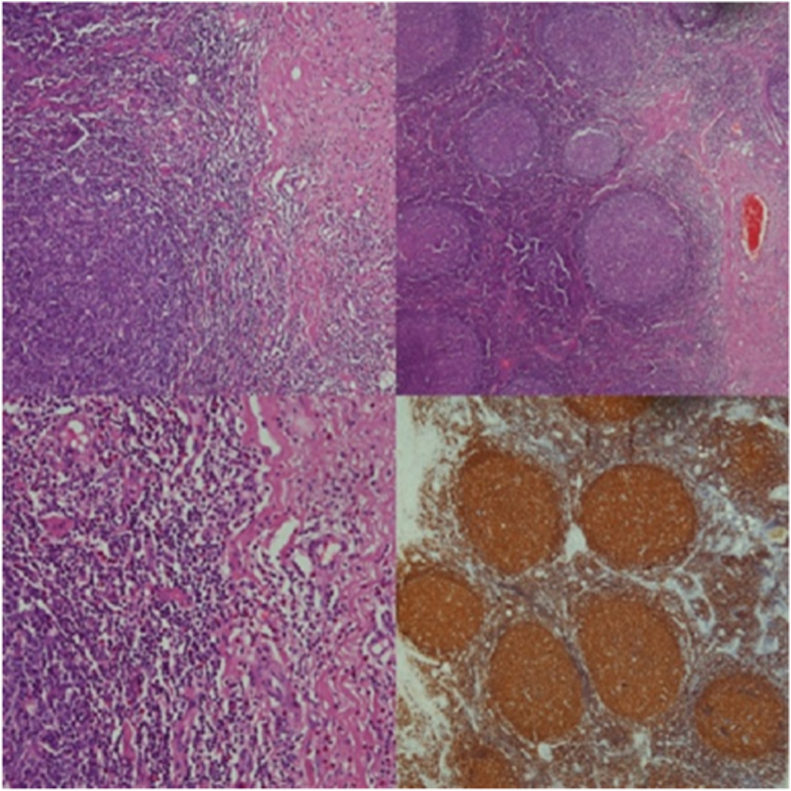
Showing histopathology features.

The patient underwent parotidectomy along with postauricular dissection. The procedure was performed by Consultant General Surgeon in a major operation theatre (OT) setting It involved a lazy “S” shaped incision and included lymph node dissection. The procedure went smoothly and had no post-op complications. The histopathology of the specimen (parotid gland) showed findings consistent with florid reactive lymphoid hyperplasia and focally increased areas of eosinophil and eosinophilic abscesses as shown in Fig. 2.

Microscopic examination showed parotid lymph nodes having variably sized lymphoid follicles with reactive germinal centers. Interfollicular areas showed increased eosinophils with many areas of eosinophilic micro abscesses (Fig. 2). Vascular component showed variable-sized congested blood vessels. On immunohistochemistry, CD3, CD20 were positive while Bcl2 was negative. All of these findings on history, examination, and histology supported the diagnosis of Kimura's disease.

**Fig. 3:**
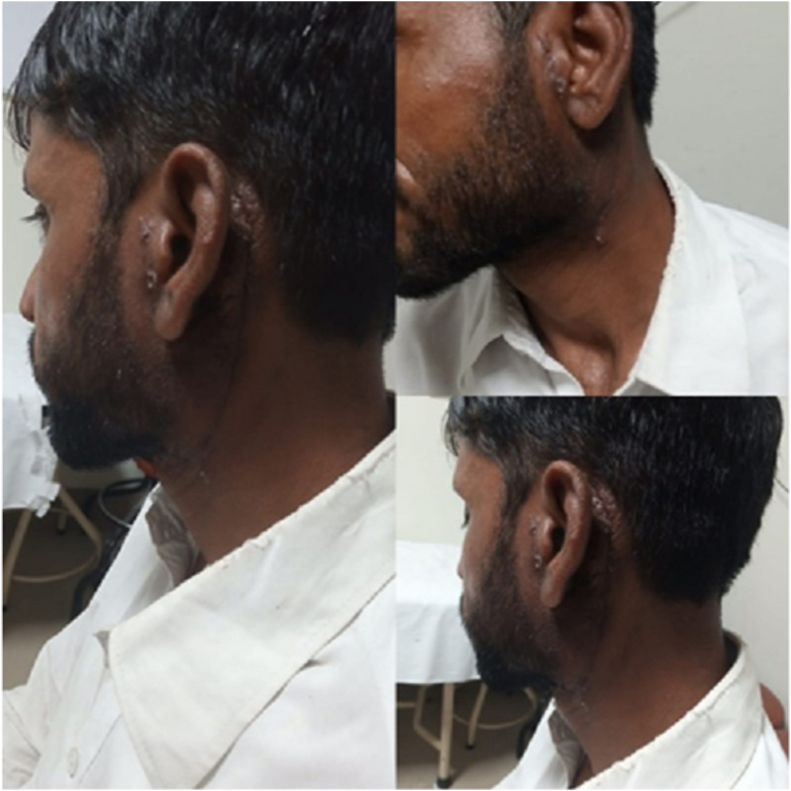
Post procedure surgical site.

No other medical treatment was given to the patient. After the surgery patient was referred for local radiation to prevent recurrence and a marked reduction in eosinophil count was reported The post-operative surgical site can be seen in Fig. 3. The post-operative investigation scan be seen in Table 2. This case report adheres to the SCARE criteria [5].

**Table 2:** Post-operative investigations.

Investigation	Results	Reference value
WBC Total	5290/μl	4000 μl–12,000 μl
RBC Total	4.34 m/μl	4.5–6.5 m/μl
Hemoglobin	12.9 g/dl	13.5–18.0g/dl
Platelet count	170,000	150,000–400,000 μl
Neutrophils	81%	54–62%
Lymphocytes	13%	25–33%
Monocytes	6%	1–4%
Eosinophils	0%	1–3%

## 3 Clinical discussion

Kimura's disease is a rare, benign noncancerous yet chronic inflammatory disorder that presents in head and neck region [[1], [2], [3], [4]]. It is mostly observed in males of Asian origin and there have been a few rare cases seen in non-Asian males [2,3]. It usually presents inthe late 20s and early 30s. No definite etiology has been determined for Kimura's disease as of yet and quite similarly no particular cause could be identified in our patient [[6], [7], [8]]. Our case also fit the diagnosis criteria, the patient was in his late 20s and of Asian origin. He had multiple swellings in the parotid region and all of them were painless as reported in the literature [[4], [5], [6], [7], [8], [9]].

Kimura's disease usually presents in the form of painless and asymptomatic subcutaneous nodules located in head and neck region [1,6,7]. The masses are mostly unilateral which grow in size overtime [10]. The presentation may vary from patient to patient but a few features are common in almost all patients [1,10]. These include marked peripheral eosinophilia, follicular hyperplasia, and increased levels of circulating IgE levels [11,12]. The triad of painless subcutaneous masses, blood and tissue eosinophilia, and markedly elevated IgE levels is the classical presentation of the disease [2]. Some of the more uncommon features include abnormalities of salivary glands and bilaterality [12]. Our patient also had peripheral eosinophilia, follicular hyperplasia, and increased IgE levels as reported in Fig. 1.

Kimura's disease is easily confused with some of the common respiratory and malignant conditions on the basis of similar history and presentation [1,[4], [5], [6]]. Some of the differential diagnoses for the condition include diseases like tuberculosis (TB), lymphomas, and angiolymphoid hyperplasia with eosinophilia (ALHE). ALHE has been confused very commonly with Kimura's disease over the years, with both being considered the same disease, but research has shown them to be two separate conditions [1,4].

For a successful diagnosis of Kimura's disease, surgical biopsy can be performed [[11], [12], [13]]. Histopathological analysis of the node or mass along with clinical findings can also assist in the decision [12]. In our patient, a similar approach was taken and Kimura's disease was confirmed on histopathologic analysis. Among radiological imaging, CT scan and Magnetic resonance imaging (MRI) can provide details regarding the size and depth of nodules [[11], [12], [13], [14]]. All of these findings on history, examination, and histology supported the diagnosis of Kimura's disease.

For treating Kimura's disease, two approaches can be taken depending upon the presentation of the patient [9,15], either with classical presentation or an asymptomatic case, a more conservative approach can be taken. The suggested treatment for Kimura's is surgical excision and was taken in our approach as well but since the lesions have a tendency for recurrence, it has to be supplemented with radiation or medical therapy [4,9,13,15]. Kimura's disease can be mediated by systemic steroids but proper compliance is required since any break or disruption in the use of drugs can result in recurrence [15]. For this reason, after discussing the patient's management plan with him he preferred the short term radiation therapy over long term steroidal approach.

## 4 Conclusion

Kimura's disease is a chronic disorder that mimics a few malignant conditions. Its diagnosis requires a thorough clinical examination supported by laboratory and radiological investigations. The disease in itself is benign and can be effectively treated by surgery coupled witha medical approach to prevent recurrence. Regardless of the approach to treatment patients with Kimura's disease usually shows a good prognosis.

## Sources of funding

N/a.

## Ethical approval

We further confirm that any aspect of the work covered in this manuscript that has involved human patients has been conducted with the ethical approval of all relevant bodies and that such approvals are acknowledged within the manuscript. IRB approval was obtained. Written consent to publish potentially identifying information, such as details or the case and photographs, was obtained from the patient(s) or their legal guardian(s).

## Consent

Written informed consent was obtained from the patient for publication of this case report and accompanying images. A copy of the written consent is available for review by the Editor-in-Chief of this journal on request.

- research registration (for case reports detailing a new surgical technique or new equipment/technology).

N/a.

## Provenance and peer review

Not commissioned, externally peer-reviewed.

## Author contributions

F.F, I.U, and I. R conceived the idea; S.W, I.U, M.J, and M.S.A collected the data; M.S.A, S.W, F.F, I. R, I.U, and M.J did write up of the manuscript; and finally, I.U, M.S.A and S.W reviewed and revised the manuscript for intellectual content critically. All authors approved the final version of the manuscript.

## Ethics statement

All ethical requirements were fulfilled before commencement of study.

## Guarantor

The Guarantor is the one or more people who accept full responsibility for the work and/or the conduct of the study, had access to the data, and controlled the decision to publish.

## Declaration of competing interest

N/a.
